# Cholera in Chicago

**Published:** 1873-09-15

**Authors:** 


CHOLERA.—There has been no general prevalence of cholera in our city during the present summer. A few cases have occurred, chiefly on the extreme southern border of the city. The general mortality from all diseases ranges a little below that which occurred during the corresponding weeks of 1872.

